# Is there a difference in regional read [^18^F]flutemetamol amyloid patterns between end-of-life subjects and those with amnestic mild cognitive impairment?

**DOI:** 10.1007/s00259-019-04282-y

**Published:** 2019-03-13

**Authors:** Gill Farrar, José Luis Molinuevo, Michelle Zanette

**Affiliations:** 1 GE Healthcare Life Sciences, Amersham, UK; 2 Barcelona Beta Brain Research Center, Pasqual Maragall Foundation and Hospital Clinic I Universitari, IDIBAPS, Barcelona, Spain; 3 GE Healthcare Life Sciences, Marlborough, MA USA

**Keywords:** Amyloid PET, [^18^F]Flutemetamol, Regional brain uptake, Autopsy, Mild cognitive impairment

## Abstract

**Purpose:**

Visual interpretation of PET [^18^F]flutemetamol images relies on systematic review of five brain regions and is considered positive when an elevated signal is observed in at least one region. Amnestic mild cognitive impairment (aMCI) is an early clinical presentation of Alzheimer’s disease (AD); hence it is of interest to determine if the pattern of visually read regional positivity between end-of-life (EoL) patients with and without dementia and aMCI patients is different.

**Methods:**

A total of 180 EoL patients with and without dementia (mean age 81 years, range 59 to 95 years) and 232 aMCI patients (mean age 71 years, range 53 to 91 years) were scanned following intravenous administration of 185–370 MBq [^18^F]flutemetamol. Images from both studies were read by two groups of five blinded readers who independently classified each of the five regions as either positive or negative. The majority interpretation made by at least three of the five readers was used as the imaging endpoint and compared with a composite standardized uptake value ratio (SUVR) analysis using a predetermined threshold.

**Results:**

Amyloid-positive images from 71 of 106 EoL patients coming to autopsy and from 97 aMCI patients were included. In the images from the EoL patients widespread deposition of amyloid was observed, with 76% of the images positive in all five regions and a further 20% positive in four regions. In the images from the aMCI patients, similar results were observed with 87% of the images positive in five regions and a further 5% positive in four regions. The mean SUVR of these positively read images was 2.24 (range 1.48 to 3.14) and 2.08 (range 1.28 to 3.04) in the autopsy and aMCI groups, respectively. There was 95.3% agreement between the visual reading and SUVR quantitation in the aMCI group and 90.4% agreement in the autopsy group.

**Conclusion:**

Patients with aMCI showed a similar distribution of amyloid deposition determined by both visual reading and SUVR to that observed in patients with and without dementia coming to autopsy. Most of the aMCI patients, who are already within the AD continuum, had widespread amyloid deposition in terms of amount and topographical progression. Attempts to observe potential initial signs of amyloid deposition should focus on populations earlier in the dementia spectrum such as patients with subjective cognitive decline or even at-risk subjects with earlier stages of disease.

## Introduction

[^18^F]Flutemetamol (Vizamyl™) is a PET imaging agent with high affinity (*K*d 6.7 nM) [1, 2] for amyloid [1, 2] whose value for the detection or exclusion of brain amyloid was studied in 831 subjects in 11 GE-sponsored clinical studies. Most of the [^18^F]flutemetamol PET signal can be attributed to neuritic amyloid plaque. In a large cohort of over 100 autopsy patients, the sensitivity and specificity of [^18^F]flutemetamol by visual reading were estimated to be approximately 90% and 91%, respectively [3, 4].

There is debate about the progressive deposition of amyloid within the brain and at what point the levels become (a) clinically significant and (b) visible for visual reading by currently approved methods described in product package inserts. A further research question remains whether visible amyloid appears in individual regions first, as described by Braak and Braak [5] (basal portions of the isocortex, followed by progressive and widespread deposition throughout the cortex), or whether it spreads across the neocortex at levels below the threshold for visual reading and can then subsequently be observed in a wider number of regions as, described by Thal et al. [6, 7].

More recently Palmqvist et al. [8] proposed that accumulation of amyloid starts in the precuneus, and medial orbitofrontal and posterior cingulate, and linked these areas to the primary regions of the default mode network. Grothe et al. [9] have described a four-stage process starting in the temporobasal and frontomedial areas where cross-sectional amyloid deposition can show a predictable regional sequence across the whole dementia spectrum. The quantitative methods used in both studies provide better sensitivity in assessing the initial stages of amyloid deposition than the currently favoured dichotomized methods of assigning brain uptake globally as either negative or positive by visual inspection. One useful application of these newer methods is likely to be for refining the inclusion criteria of subjects with early disease included in therapeutic intervention studies.

The post hoc analysis presented here had three main aims: first, to assess the extent of regional visual read positivity in both an end-of-life (EoL) population (comprising patients both with and without dementia) and a younger group of patients with amnestic mild cognitive impairment (aMCI); second, to refine image reading methodology with this knowledge; and third to compare the majority read results from each cohort with a quantitative measure (standardized uptake value ratio, SUVR) to examine the consistency of visual reading in both populations. The data generated in this study could become valuable as the Alzheimer’s disease (AD) spectrum is now recognized as ranging from those with overt clinical symptoms to those with less well-defined disease such as early aMCI and subjective cognitive decline [10], and hence it is important to define the accuracy and utility of amyloid PET imaging in these cases.

## Materials and methods

### Clinical cohorts used for regional read analysis

Demographic details of the two clinical cohorts imaged with [^18^F]flutemetamol and used for the regional read analysis are presented in Tables 1 and 2. Details common to the study of both cohorts were the intravenous administration of [^18^F]flutemetamol (Vizamyl) followed by the acquisition of PET images, and the blinded reading of the PET images.

**Table 1:** Demographic characteristics of the 106 patients who came to autopsy of 180 initially imaged

Characteristic	Value
PET-positive, *n* (%)	71 (59)
Age (years), mean (range)	81 (59–95)
Gender, *n*	
Male	48
Female	58
Clinical history as reported in case notes, *n*	
Alzheimer’s disease	53
Other dementia	25
No dementia reported in case notes	27
Unspecified memory loss	1
Mean time to death (months)	7.5
Time from scan to death (years), *n* (%)	
<1	82 (77)
>1 to <2	19 (18)
>2 to <3	5 (5)

More comprehensive study details can be found in Ikonomovic et al. [3] and Salloway et al. [4]

**Table 2:** Demographic characteristics of the 232 aMCI patients (clinically diagnosed using the Petersen criteria [11]) followed up for 3 years after [^18^F]flutemetamol PET imaging

Characteristic	Value
Reviewed by a CAC	224
Age (years), mean (range)	71 (53–91)
Gender, *n* (%)	
Male	114 (49)
Female	118 (51)
Clinical assessments	Every 6 months for 3 years
Endpoint (assessed on-site, confirmed by CAC)	Conversion to clinically probable AD
Amyloid scan, *n* (%)^a^	
Positive	97 (43)
Negative	127 (57)
Conversion from aMCI to probable AD, *n* (%)	84 (36)
Hazard ratio for conversion at 3 years after positive/negative scan	2.5

More comprehensive study details can be found in Wolk et al. [11]

*CAC* Clinical Adjudication Committee

^a^Of those reviewed by the CAC

The first clinical cohort was an autopsy EoL population previously described in detail by Ikonomovic et al. [3] and Salloway et al. [4]. Of 180 patients previously imaged after administration of 185–370 MBq [^18^F]flutemetamol, 106 (mean age 81 years, range 59–95 years) came to autopsy with a mean time from imaging to death of 7.5 months (Table 1). Of the 106 EoL patients who came to autopsy, 105 were reportable (1 did not have a visual read of PET images). Of the 105 evaluable scans, 71 were read as positive (abnormal) and 34 as negative (normal; Table 3 shows a breakdown of the scan results).

**Table 3:** Clinical diagnosis in the 105 evaluable end-of-life patients who came to autopsy in relation to [^18^F]flutemetamol PET imaging results (amyloid-positive and amyloid-negative)

Clinical diagnosis^a^	Positive (abnormal) (*N* = 71)	Negative (normal) (*N* = 34)	Total (*N* = 105)
AD	42 (79.2%)	11 (20.8%)	53 (50.5%)
Other dementing disorder	19 (79.2%)	5 (20.8%)	24 (22.9%)
MCI	0 (0%)	0 (0%)	0 (0%)
None	10 (37%%)	17 (63%)	27 (25.7%)
Memory loss (unspecified)	0 (0%)	1 (100%)	1 (1.0%)

^a^As reported in the case notes

The aMCI patients were, on average, 10 years younger than the EoL patients (mean age 71 years, range 53–91 years) and comprised 232 patients who took part in a 3-year follow-up study to determine whether a positive [^18^F]flutemetamol PET scan or positivity using other biomarkers would increase the risk of progression to AD (Table 2). The demographics and inclusion criteria for this study have been described by Wolk et al. [11]. Of the 232 aMCI patients, 224 were reviewed by a Clinical Adjudication Committee (CAC) to assess conversion status [11]. Of the 224 aMCI patients with evaluable images, 97 showed a positive read (i.e. positive in any one of the regions listed in section Image reading methodology below), and the original case report forms collected for the blinded read were further analysed to examine regional read positivity. The regional read in patients with negative scans were not further assessed.

The overall image read results, including both positive and negative image reads, are reported in the section Overall positive and negative image read results. Only those scans with an overall positive read from the majority of readers (at least three of five) were considered in the regional read analysis.

### Image reading methodology

**Fig. 1:**
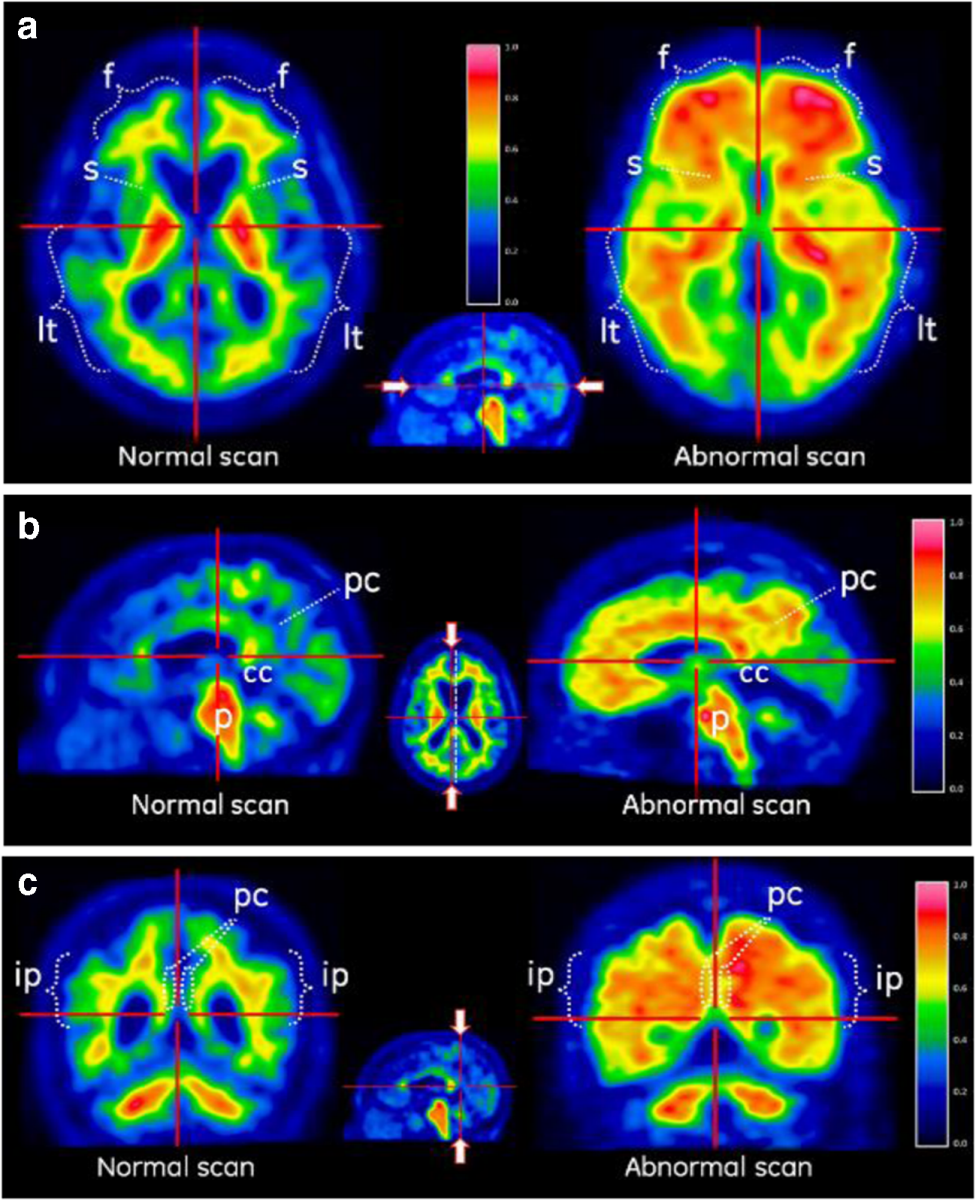
Example [^18^F]flutemetamol PET images interpreted as normal and abnormal. **a** Axial images. *Left* The normal (negative) image shows a sulcal/gyral white matter pattern in the frontal (*f*) and lateral temporal (*lt*) regions, as well as high uptake in the thalamus. *Right* The abnormal (positive) image does not show this pattern due to grey matter uptake of [^18^F]flutemetamol. In these cortical regions, the intensity is higher in the abnormal (positive) image (>60% of maximum) than in the normal image (<60% of maximum), and in the abnormal image the intensity radiates to a sharply defined convex edge, but in the normal image the intensity tapers to the periphery of the tissue in these regions. Striatal gaps (*s*) between the thalamus and frontal white matter are visible in the normal image but not in the abnormal image as [^18^F]flutemetamol shows consistent binding to striatal amyloid [12]. **b** Parasagittal images touching the medial surface of one hemisphere. *Right* The abnormal image shows increased tracer uptake (>60% of maximum) in the posterior cingulate/precuneal (*pc*) region, superior and posterior to the corpus callosum (*cc*). *Left* The normal image shows <60% of maximum intensity in the same regions. The pons (*p*) shows high intensity in this sagittal view (approximately 90% of maximum). **c** Coronal images (slices posterior to the corpus callosum). *Left* Normal images show a white matter sulcal/gyral pattern. *Right* the abnormal image loses this pattern and also shows increased uptake in the posterior cinguli (*pc*) and an increased radial extent of high intensity uptake to the lateral surfaces of the temporoparietal lobes as well as the insula region. The inferior parietal (*ip*) region in particular shows increased intensity and is a robust read region as is less susceptible to atrophy [12]

Ten Board-Certified readers (five for each study cohort) were trained using either an electronic training programme [12] for the autopsy cohort, or in-person training [13]. Both methods were comparable in terms of image read methodology and all readers passed the same test of competence in assessing images before performing the image reads. Each reader recorded the frontal, lateral temporal, temporoparietal/insula and posterior cingulate/precuneus and additionally the striatum (read region specific to [^18^F]flutemetamol) as positive or negative for amyloid, and recorded the overall amyloid status. A scan was deemed positive if there was uptake in any one region. A scan was deemed negative if there was no uptake in all five regions. Example scans interpreted as abnormal and normal are shown in Fig. 1.

### Comparison of visual and quantitative SUVR results

For each cohort (EoL and aMCI) both negative and positive visual reads were compared with the SUVR values. Measurement of SUVR was based on the method described by Vandenberghe et al. [14]. In brief, summed PET images were spatially normalized into standard space by defining volumes of interest for various regions on the PET images using an automated anatomical atlas. A composite cortical SUVR based on the combined uptake in the lateral frontal cortex, lateral temporal cortex, parietal cortex, anterior cingulate and posterior cingulate was measured using the cerebellar cortex as the reference region. The SUVR threshold was taken as 1.56 as previously defined by Vandenberghe et al. [14]. These authors describe in detail the derivation of this threshold as the midpoint between the mean SUVR in AD patients and the SUVR in a population of elderly healthy volunteers [14].

### Statistics

The majority read data (the decision from at least three of five blinded readers; brain region either positive or negative) were used as the output data in this analysis. Data from each of the two population groups were tabulated in two ways: first as the percentage of the samples positive in all five read regions followed by the percentages positive in four, three, two or one region, and second as the percentage of samples positive in a particular region (e.g. frontal). Descriptive analysis of the data where appropriate was adequate for the assessment of the regional reads of the positive scans in both cohorts, as well as for the descriptions of the read characteristics of the clinical cases and the comparisons between visual and quantitative SUVR measures. Additionally, the Mantel-Haenszel test was used to assess the association between the two populations studied and the patterns of regional amyloid positivity.

## Results

### Overall positive and negative image read results

In the original autopsy studies [3, 4], 42 of 53 AD patients (79%) had a positive amyloid scan by majority read and 11 of 53 (21%) had a negative scan. A similar ratio of positive/negative scans was found among patients with other dementing disorders, and 17 of 27 patients (63%) with no cognitive symptoms had a negative scan. Of the ten normal subjects without a cognitive deficit and a positive scan, three were older than 72 years (72, 73 and 74 years), one was 79 years old and six were older than 82 years (range 82–95 years).

Of the 224 aMCI patients, 97 (43%) were reported as overall positive. The mean age of patients with a positive scan was 73 years (range 53 to 91 years) and the mean age of those with a negative scan was 70 years (range 55 to 90 years). The amyloid positivity in this MCI population was 43%. This is slightly lower than the positivity rate of 48.7% (range 44.5% to 53%) found by Jansen et al. [15] in a meta-analysis in patients with a similar average age (70 years).

### Regional read analysis in patients with a positive scan

#### End-of-life population

A descriptive analysis of the regions studied in the EoL population is presented in Table 4. Unsurprisingly, 96% of the EoL patients showed positivity in four or all five regions. All five regions were positive in 54 of the 71 patients: 33 with AD (61%), 15 with other dementing disorders (28%), and 6 with no cognitive decline (11%). Among the 17 patients showing positivity in fewer than the full five regions, the proportions were similar even though the numbers were small: 9 with AD (53%), 4 with other dementing disorders (23.5%), and 4 with no cognitive decline (23.5%). Only three patients showed positivity in three or fewer regions, and all these patients were older than the mean age of 81 years: three regions in a 93-year-old with another dementia, two regions in a 91-year-old with AD, and one region in an 84-year-old with AD. Thus in the EoL population there were minimal differences in the regional read patterns among those with AD, those with other dementias and those with no cognitive decline. The posterior cingulate was positive in 100% of the EoL patients, followed by the temporoparietal region (in 96%), the frontal region (in 93%), the striatal region (in 92%), and the lateral temporal lobes (in 87%).

**Table 4:**
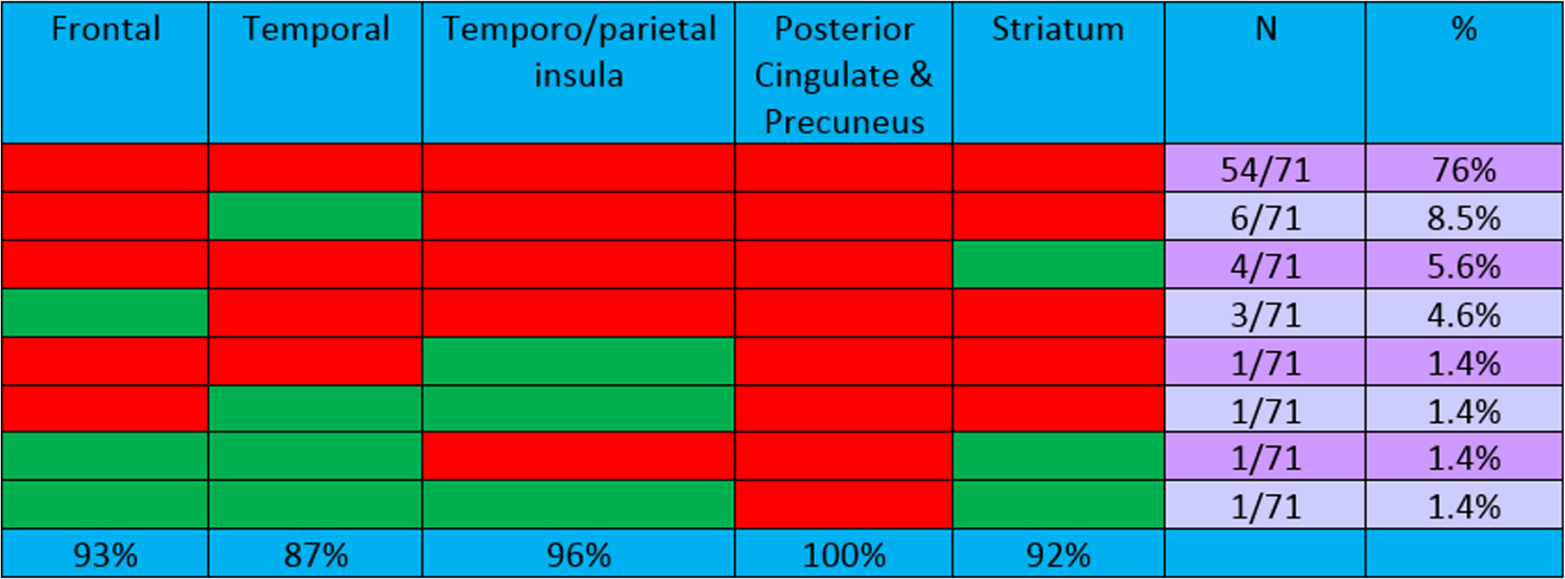
Combinations of regions positive (red) for [^18^F]flutemetamol: autopsy study (71 + ve cases)

Additional measurements comparing reader consistency with regional CERAD pathology reported at autopsy (frontal 92% consistency, temporal 84%, parietal 91%, posterior cingulate/precuneus 93%) are included in the detailed neuropathology report in these autopsy cases [3].

#### Amnestic MCI population

A descriptive analysis of the regions studied in the aMCI population is presented in Table 5. This population was on average 10 years younger, and showed a comparable pattern, with 87% of the patients showing positivity in all five regions and a further 5% showing positivity in four regions. Only 8 of 97 aMCI patients showed limited positivity in three, two or one region only. In the aMCI patients, 97–98% showed positivity in both the posterior cingulate and the striatum (the regions most commonly affected) with lower percentages showing positivity in the other regions (93% frontal and temporal, 91% temporoparietal). The aMCI patients were also subdivided into those with early and those with late MCI on the basis of logical memory scores (as described by Wolk et al. [11]). Among 13 of the 97 patients showing positivity in only four or fewer regions there was little difference (even though the numbers were small) between seven patients with early MCI and six patients with later MCI. Comparing the EoL and aMCI populations, the posterior cingulate was the region most commonly positive in both populations, with 100% and 97% of patients showing positivity in this area.

**Table 5:**
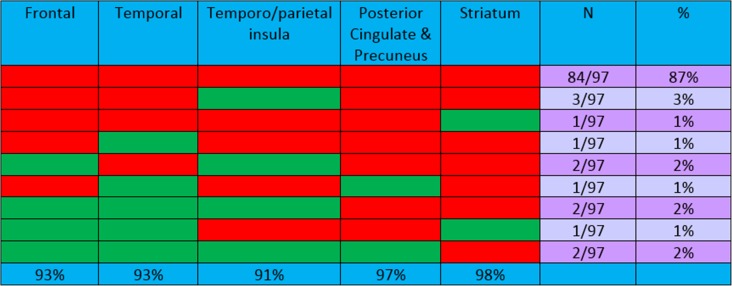
Combinations of regions positive (red) for [^18^F]flutemetamol: aMCI study (97 + ve cases)

#### Comparative analysis of the EoL and aMCI populations

The aim of the analysis was to test if there was an association between patient population and the number of positive regions. The number of positive regions was considered an ordinal variable with an increased number of positive regions indicating more severe disease. The Mantel-Haenszel test indicated that there was no association between patient population and the number of positive regions (*p* = 0.7742). Therefore, there is not enough evidence to suggest that regional amyloid uptake was different between the patient populations.

#### Majority read versus SUVR data

SUVR and majority read data were available for 104 of the 106 patients who came to autopsy. The mean composite SUVR in 34 patients negative/normal by majority read was 1.40 (range 1.01 to 2.04) and in 70 patients positive/abnormal by majority read was 2.24 (range 1.48 to 3.14). In the 232 aMCI patients, the mean composite SUVR in 134 patients negative by majority read was 1.25 (range 0.95 to 1.72) and in 98 patients positive by majority read was 2.08 (range 1.28 to 3.04). The majority read results from both autopsy and aMCI patients were then compared with the SUVR results with the patients dichotomized according to the SUVR threshold of 1.56 (>1.56 considered abnormal, ≤1.56 considered normal [14]). The rates of agreement were 90.4% and 95.3% in the autopsy patients (94/104) and aMCI patients (221/232), respectively.

In ten autopsy patients the read and SUVR results were discordant: in eight patients the read was false-negative and in two false-positive. The patients with false-negative reads showed SUVR close to the 1.56 threshold with composite SUVR ranging from 1.57 to 2.04 and all had extensive atrophy which could have led to the read being assessed as negative when the quantitative assessment showed evidence of positive [^18^F]flutemetamol uptake. The two patients with false-positive reads showed SUVR just below the threshold of 1.56 (SUVR 1.483 and 1.476, respectively).

The aMCI patients had a higher rate of agreement between the visual read and SUVR results than the autopsy patients. In 11 of the 232 aMCI patients the read and SUVR results were discordant: in four patients the read was false-negative and in seven false-positive. In most of the aMCI patients with discordance, SUVR was close to the 1.56 threshold. In patients with a false-negative read the composite SUVR ranged from 1.574 to 1.721 and in those with a false-positive read from 1.276 to 1.560.

## Discussion

In this study aMCI patients showed a comparable distribution of amyloid deposition as measured by both regional visual read and quantitative SUVR to that observed in the EoL autopsy patients. Most of the aMCI patients, who were already on the AD continuum, had widespread amyloid deposition in terms of amount and topographical progression. In both cohorts there were a few patients with amyloid positivity in a limited number of regions, and therefore it is important in visual image reading to systematically assess each of the five regions outlined in the [^18^F]flutemetamol image interpretation instructions so as not to miss such cases. The image read results were also compared with quantitative SUVR results to assess the concordance between the approved method of visual image assessment which is routinely used clinically and quantitation which is more widely used in the research setting, particularly where it can be used to help resolve an equivocal image interpretation.

Since aMCI is one of the main clinical presentations assessed in practice, the information has practical use for routine image interpretation. Currently the methods endorsed by regulations for assigning a brain as positive or negative for amyloid are via visual reading only. Results from this analysis indicate that, even in this early phase of AD, the deposition of amyloid is widespread in terms of amount and topographical progression and is consistent with the hypothesis of Villemagne et al. [16] that amyloid deposition occurs early and extensively in the AD spectrum years before the onset of major clinical symptoms. The ability to see the earliest signs of amyloid deposition would have to focus on less demented populations in the AD continuum such as those with subjective cognitive decline or those at risk even earlier in the disease course.

Additionally, some interesting observations were made in this analysis. Among 53 patients with an AD diagnosis recorded in the case notes, 42 (79%) showed [^18^F]flutemetamol positivity and 21% of AD patients diagnosed with AD had a negative scan (at autopsy a number of other pathologies were recorded, for example Lewy body dementia, progressive supranuclear palsy, vascular dementia and cerebral amyloid angiopathy; individual cases are discussed in [3]). One explanation for this observation is that there is some inaccuracy in the clinical diagnosis of AD which may have been performed without biomarker guidance. In a larger cohort analysis, Beach et al. [17] similarly found discordance between a clinical diagnosis of AD and an absence of amyloid pathology at autopsy in up to 30% of patients. Additionally, in a recent meta-analysis Fantoni et al. [18] found that just over 30% of patients with a diagnosis of AD on presentation had AD ruled out by a negative amyloid PET result, and a similar figure of 29% was found by Barthel and Sabri [19]. These findings indicate that the work-up of the AD patient could be improved by the presence of biomarker data in the form of an amyloid PET scan [20].

Furthermore, a high percentage of patients had no evidence of cognitive disorder but did show a positive amyloid scan. These subjects were generally more than 80 years old, which is consistent with the high prevalence of amyloid in the very elderly as observed by Rowe et al. [21] and summarized by Jansen et al. [15]. An amyloid scan to support a clinical diagnosis (in particular AD rule-in) may therefore be more useful in patients showing evidence of cognitive impairment and who are younger than 80 years as per the appropriate use guidelines (one of the use categories is in early age onset dementia) [22], though ruling out AD in an older negative case would have considerable value too.

Clinical staging of AD may be possible by applying the staging criteria for amyloid pathology progression of Thal et al. [6]. Here the stage between preclinical and clinical AD is characterized by the involvement of neocortical amyloid and the additional presence of amyloid in the striatum (Thal stage 3). In our population of 97 aMCI patients, only two showed no [^18^F]flutemetamol uptake in the striatum in the presence of positive uptake in other neocortical regions, so it is surmised that the hypothesis of Thal et al. would need to be tested in cohorts earlier in the AD spectrum than those in this study. Indeed, Hanseeuw et al. [23] investigated PET staging in 1,433 patients from both the Alzheimer’s Disease Neuroimaging Initiative (ADNI) and Harvard Aging Brain Study (HABS) and found that 29% (ADNI) and 12% (HABS) of the MCI patients had a negative striatal PET scan in the presence of a positive neocortical scan, pointing to a larger number of patients in this interim phase (Thal stage 3) between preclinical and clinical AD. Grothe et al. [9] have also proposed a four-stage model for characterising the systematic deposition of amyloid visualized on PET. Both models (Hanseeuw et al. [23] and Grothe et al. [9]) appear to correlate well with cognitive decline across the dementia spectrum. The primary difference between these recent models and those of Thal et al. are that Hanseeuw et al. and Grothe et al. assessed more specifically the regional cortical stages which Thal et al. include in a higher level in their stages 1–3.

The inclusion of the striatum in the staging models of Thal et al. [6], Hanseeuw et al. [23] and Grothe et al. [9] is also interesting to note, since the work of Beach et al. [24] indicates that [^18^F]flutemetamol binds to diffuse amyloid which is the primary form of amyloid in this region. Amyloid fibrils in diffuse amyloid are less dense and hence the striatum may be an area that may show earlier signs of therapeutic efficacy when the treatment includes an antiamyloid component. A population with no striatal involvement (but cortical amyloid) may also be interesting to study for immediate conversion (and therapy intervention).

Incidental cases of amyloid positivity, for example in the striatum only (2 of 97 aMCI patients, Table 6), may have been due to other less frequent clinical phenotypes such a presenilin mutation [25], although the presence of atrophy confounding the regional reads in neocortical areas also need to be considered. It is not known whether the 2 of 97 patients in this analysis had a presenilin mutation.

**Table 6:** Number of amyloid-positive regions in patients of both study groups based on majority read by three of five readers

Study group	Number of amyloid-positive regions^a^					Total	*p* value^b^
	1	2	3	4	5		
aMCI	2 (2.1%)	3 (3.1%)	3 (3.1%)	5 (5.2%)	84 (86.6%)	97	0.7742
Autopsy	1 (1.4%)	1 (1.4%)	1 (1.4%)	14 (19.7%)	54 (76.1%)	71	
Total	3	4	4	19	138	168	

^a^Regions include frontal, temporal, temporoparietal/insula, posterior cingulate and precuneus, and the striatum

^b^Based on the Mantel-Haenszel test

The results of this regional analysis also have implications for the visual reading of [^18^F]flutemetamol PET scans. The result of this post hoc analysis could be used to refine the training guidance given to physicians who are learning to read [^18^F]flutemetamol PET scans. For example, it is important to note that readers should not assume that all cases are either positive or negative in all five regions, but a small proportion of cases will show positivity in three, two or one region. Therefore, a systematic review of all five regions would be recommended to assign a case as either positive or negative.

The posterior cingulate/precuneal region has been found to be the most robust region for the visual reading of [^18^F]flutemetamol PET scans, probably because the medial surfaces seem less prone to atrophy and also the two regions in each hemisphere are proximally located. The striatum is also an easy region to read and is specifically and uniquely highlighted in the read interpretation instructions for [^18^F]flutemetamol as it is less susceptible to atrophy than other regions. Here the amyloid pathology is primarily in the diffuse form [24] and the striatum is a region of interest for Thal phasing as discussed above. Of the five regions inspected in the reading of [^18^F]flutemetamol PET scans in an overall positive read, the lateral temporal lobes are read as negative most frequently. Despite being one of the original regions for CERAD, the lobes are sometimes affected by atrophy and also have the added problems of partial volume issues because of the presence of CSF and the fact that the recommended axial read plane (AC–PC plane) is not directly perpendicular to the horizontal axis of the temporal lobe. In this case, the use of anatomical CT or MRI is recommended to assist in the reading, although the lateral temporal lobes should not be considered in isolation.

A limitation of this work is that the reading results presented here were derived from populations that are seen later in the dementia spectrum, that is aMCI and EoL patients. The EoL patients studied were originally used for measurement of the performance (sensitivity/specificity) of the tracer and included a heterogenous group of patients in whom the diagnosis was spontaneously reported in the case notes, whereas the aMCI patients were included via the consistent use of diagnostic criteria [11]. The training methodology for the visual reading of [^18^F]flutemetamol PET scans was validated using cases from these studies [12]. More recent studies are now focusing on patients earlier in the dementia spectrum such as those with subjective cognitive decline and preclinical at-risk patients, as targeted populations for early intervention. Visual inspection methods may, therefore, need supplementing with supporting quantitative analysis [26] to increase the sensitivity in detecting early pathological changes just below the standard thresholds [27] and to improve the accuracy of reading when the cases are close to the pathological threshold where reader confidence is lower [3] or when the reader is less experienced in amyloid PET reading or does not interpret these images on a routine basis.

In summary, [^18^F]flutemetamol showed a widespread topographical presence in a clinically relevant aMCI cohort as well as in a validation cohort of EoL patients. The accuracy of the tracer is high and provides a robust measure of brain amyloid that is particularly relevant for the assessment of the pathological continuum which underpins diagnostic criteria in both clinical practice and in future AD research frameworks [28].
